# Cosmetic enhancement as social signaling: context-dependent effects on facial evaluation in China and the United Kingdom

**DOI:** 10.3389/fpsyg.2026.1872745

**Published:** 2026-08-06

**Authors:** Fuqun Liang, Jianbing Li, Hao Hong, Siyue Zhang, Yuanlin Yu, Xianyou He, Anthony C. Little

**Affiliations:** 1 School of Psychology and Entrepreneurship Education, Guangdong University of Finance, Guangzhou, China; 2 Department of Psychiatry, The Affiliated Guangdong Second Provincial General Hospital of Jinan University, Guangzhou, China; 3 School of Psychology, South China Normal University, Guangzhou, China; 4 Department of Psychology, University of Bath, Bath, United Kingdom

**Keywords:** makeup, cross-culture, attractiveness, trustworthiness, dominance

## Abstract

**Introduction:**

Human observers rapidly infer socially relevant traits from faces, yet these judgments can be altered by appearance modifications such as cosmetic enhancement. It remains unclear whether cosmetic enhancement produces similar effects across different dimensions of facial evaluation and whether these effects are interpreted consistently across cultural contexts. The present study examined how cosmetic enhancement influences facial social evaluation among Chinese and British perceivers.

**Methods:**

In a preregistered mixed-design experiment, 100 Chinese participants and 99 British participants evaluated East Asian and Caucasian female faces presented in natural, light-makeup, or heavy-makeup conditions. Participants rated each face on attractiveness, dominance, competence, youthfulness, and trustworthiness.

**Results:**

Cosmetic enhancement increased perceived attractiveness and youthfulness and also enhanced competence and dominance overall. However, several effects varied according to perceiver nationality and target ethnicity. Trustworthiness showed a particularly context-dependent pattern. Chinese participants rated lightly made-up faces as more trustworthy than natural or heavily made-up faces, whereas British participants showed no significant differences in trustworthiness across makeup conditions. Additional interactions indicated that the effects of cosmetic enhancement on dominance, competence, and youthfulness also depended on the combination of perceiver nationality and target ethnicity.

**Discussion:**

These findings suggest that makeup-induced appearance cues can operate as social signals in facial impression formation. Cosmetic enhancement produced relatively general benefits for beauty- and vitality-related impressions, whereas its effects on status-, agency-, and trust-related judgments were more dependent on makeup intensity and the cultural and intergroup context in which the face was evaluated.

## Introduction

Human observers rapidly extract socially consequential information from faces. Within a fraction of a second, individuals form impressions of trustworthiness, dominance, and attractiveness, and these inferences are remarkably consistent across perceivers (Todorov et al., 2015; Willis and Todorov, 2006). Such rapid trait attribution is widely understood as an adaptive feature of human cognition, enabling the evaluation of potential allies, competitors, and mates under conditions of uncertainty (Oosterhof and Todorov, 2008; Todorov et al., 2008).

Yet faces are not encountered only as unmodified biological signals. In daily social life, people actively shape their appearance in ways that change the facial cues available to observers (Kowal et al., 2022). Among these practices, cosmetic use is one of the most widespread and socially visible forms of appearance enhancement (Kowal et al., 2022). Its psychological significance lies not only in altering how a face looks, but also in changing the social information that observers may infer from the face (Davis and Arnocky, 2022; Batres et al., 2025). In this sense, cosmetic enhancement can be understood as a form of social signaling in facial impression formation, whereby makeup-induced appearance cues shape judgments of socially relevant traits (He et al., 2023a,b; Liang et al., 2025).

From an evolutionary perspective, cosmetic enhancement may increase the salience of facial cues that observers use when forming impressions of attractiveness and youthfulness. Recent work further suggests that makeup enhances attractiveness by modifying multiple known factors of facial beauty, including perceived age, femininity, averageness, symmetry, and health-related cues (Batres et al., 2025). Related evidence also indicates that increasing facial skin homogeneity can enhance attractiveness even when observers do not consciously detect that the face has changed (Sun et al., 2022).

Empirical research consistently demonstrates that faces with cosmetics are rated as more attractive and, in some contexts, more competent and socially dominant (Aguinaldo and Peissig, 2021; Klatt et al., 2016; Mileva et al., 2016). Recent empirical work has further shown that the effects of appearance enhancement extend beyond attractiveness. Beauty filters in dating profiles, for example, can increase perceived attractiveness and dating intention while reducing perceived trustworthiness (Appel et al., 2023). Similarly, beautification strategies can shape trustworthiness and morality judgments, with these effects moderated by evaluator and target characteristics (He et al., 2023a,b; Liang et al., 2025).

These findings suggest that cosmetic enhancement may shape observer-based impressions that are relevant to interpersonal evaluation, including attractiveness, youthfulness, competence, and dominance (Batres et al., 2025; Mileva et al., 2016). Such impressions are important because they are often linked to broader social judgments concerning desirability, capability, and social presence (Lin et al., 2025). However, the same appearance cues that enhance positive impressions may also change how observers interpret the person behind the face (Liang et al., 2025). When enhancement is perceived as highly deliberate or artificial, it may introduce ambiguity in judgments of authenticity, morality or trustworthiness (Samper et al., 2018; Liang et al., 2025).

Indeed, evidence suggests that cosmetic enhancement does not uniformly improve social evaluation (He et al., 2023a,b; Kuehl et al., 2018). While cosmetics often increase perceived attractiveness and competence, they may simultaneously reduce perceived authenticity, morality and trustworthiness (Bernard et al., 2020; Mittal and Silvera, 2020; Samper et al., 2018; Liang et al., 2025). This pattern suggests that cosmetic enhancement may have differentiated effects across dimensions of social perception. It may strengthen attractiveness- and status-related impressions while producing more context-dependent consequences for broader interpersonal and moral evaluations.

From a social-evolutionary perspective, facial evaluation often involves multiple dimensions that do not necessarily align. Appearance-based judgments such as attractiveness and youthfulness may not always correspond to trust-related judgments such as perceived trustworthiness or long-term cooperative value. Contemporary models of face perception suggest that social evaluation from faces is organized around a small number of functional dimensions. The influential valence–dominance model highlights two central dimensions of facial evaluation: valence-related judgments, such as trustworthiness, and dominance-related judgments, such as perceived power or formidability (Oosterhof and Todorov, 2008; Todorov et al., 2015).

However, the structure of facial evaluation may not be fully universal across cultural contexts. Cross-cultural work has shown that although a valence-related dimension often emerges across perceiver groups, Chinese perceivers’ social judgments of faces may be less strongly organized around dominance and more strongly characterized by capability- or competence-related impressions (Sutherland et al., 2018; Wang et al., 2019). This suggests that dominance and competence should both be considered when examining facial evaluation across Chinese and British perceivers. In the context of cosmetic enhancement, appearance-based judgments such as attractiveness and youthfulness are also central, because beautification directly modifies cues associated with beauty, vitality, and age-related impressions. Cosmetic enhancement may therefore affect multiple domains of facial evaluation, and these domains may not change in parallel.

If cosmetics operate as social signals in facial impression formation, their effects should depend not only on the appearance cues themselves but also on the cultural context in which observers interpret them (He et al., 2023a,b; James et al., 2018). Signal perception is shaped by local social environments, appearance norms, and intergroup contexts. For example, observers may interpret enhanced appearance differently when evaluating in-group versus out-group targets, as intergroup contexts can heighten vigilance and alter weighting of dominance and trust cues (Vorauer and Turpie, 2004).

Cross-cultural variation provides a natural context for examining such calibration. While the biological substrates of facial evaluation are shared across populations, the weighting of attractiveness, dominance, and trust cues may vary as a function of social norms, exposure, and ecological conditions (Henrich et al., 2010). If cosmetic enhancement operates as a social signal in facial impression formation, its evaluative consequences may differ depending on perceiver population and target ethnicity. Importantly, such variation need not imply fundamentally different perceptual mechanisms. Rather, it may reflect differences in how observers weight the benefits and costs associated with enhanced appearance.

### The present study

The preceding review suggests that cosmetic enhancement can be understood as a form of social signaling in facial impression formation. The central question is whether cosmetic enhancement carries a relatively stable social meaning or whether its meaning depends on the trait being judged and the cultural context in which it is interpreted. Existing work has shown that makeup often increases perceived attractiveness and can also influence judgments such as competence, dominance, and trustworthiness. Yet it remains unclear which evaluative consequences of cosmetic enhancement are relatively general across perceivers and faces, and which are more sensitive to makeup intensity, perceiver cultural background, and target ethnicity.

The present study addressed this question by examining five dimensions of facial evaluation that capture three theoretically relevant domains. Attractiveness and youthfulness were included as beauty- and vitality-related impressions that are central to cosmetic enhancement and may show relatively general enhancement effects. Dominance and competence were included as status- and agency-related impressions, because these judgments may be shaped by culturally variable frameworks of facial evaluation. Trustworthiness was included as a trust-related judgment, because it may depend more strongly on whether cosmetic enhancement is interpreted as socially appropriate, natural, or overly deliberate. This set of outcomes allowed us to examine whether makeup-induced appearance cues produce broad enhancement effects across facial evaluation or more differentiated effects across beauty/vitality-, status/agency-, and trust-related domains.

To test these questions, we conducted a preregistered mixed-design experiment in which Chinese and British participants evaluated East Asian and Caucasian female faces presented in three makeup conditions, including natural, light makeup, and heavy makeup. This design allowed us to examine whether cosmetic enhancement systematically shifts facial evaluation and whether these effects vary depending on makeup intensity, perceiver cultural background, and target ethnicity.

Based on the preceding theoretical review and empirical evidence, we proposed the following hypotheses.

*Hypothesis 1*: Faces with cosmetic enhancement, including light or heavy makeup, will be rated as more attractive, youthful, competent, and dominant than natural faces.

*Hypothesis 2*: The effects of cosmetic enhancement on trustworthiness judgments will be more context-dependent than its effects on attractiveness-, youthfulness-, competence-, and dominance-related judgments, varying as a function of makeup intensity and perceiver cultural background.

## Methods

### Participants

*A priori* power analysis was conducted using G*Power 3.1.9 (Faul et al., 2009), based on previous research (Mileva et al., 2016), with parameters set at *f* = 0.25 and *α* = 0.05. The analysis indicated that a sample of 172 participants would be required to achieve high statistical power (1 − *β* = 0.95). To account for potential data exclusions, a total of 200 participants were recruited for the present study. All participants had normal or corrected-to-normal vision and reported no extended exposure to other cultures.

Chinese participants were recruited via the Credamo, with an initial sample of 110. Ten participants were excluded due to failure on attention checks, resulting in a final sample of 100 participants (47 men, 53 women; *M*_age_ = 31.40, SD = 10.81). British Caucasian participants were recruited via Prolific, with an initial sample of 135. Twenty-nine participants were excluded for failing attention checks, and six were excluded for incomplete task completion, yielding a final sample of 99 participants (47 men, 52 women; *M*_age_ = 36.13, SD = 12.45). The study protocol was preregistered on the OSF platform^1^ and approved by the Ethics Committee at the University of Bath.

### Experimental design

The present study employed a 2 (participant cultural background: Chinese| British; between-subjects) × 2 (face type: East Asian|Caucasian; within-subjects) × 3 (Makeup level: natural|light makeup|heavy makeup; within-subjects) mixed factorial design. Participants were categorized based on cultural background if they had lived in China or the United Kingdom for more than 10 years, with no more than 1 year of residence in other cultural contexts.

Makeup level was manipulated using the Meitu app^2^, which generated light and heavy makeup conditions based on the original natural faces. Dependent variables included perceived attractiveness (“How attractive do you feel this face is?”), perceived trustworthiness (“How trustworthy do you think this person is?”) (He et al., 2023a,b), perceived competence (“How capable do you think this person is?”), perceived youthfulness (“How youthful does this person appear to be?”), and perceived dominance (“How dominant do you think this person appears?”). All ratings were made on a 7-point Likert scale, with 1 indicating a completely negative evaluation and 7 indicating a completely positive evaluation.

### Stimuli

**Figure 1:**
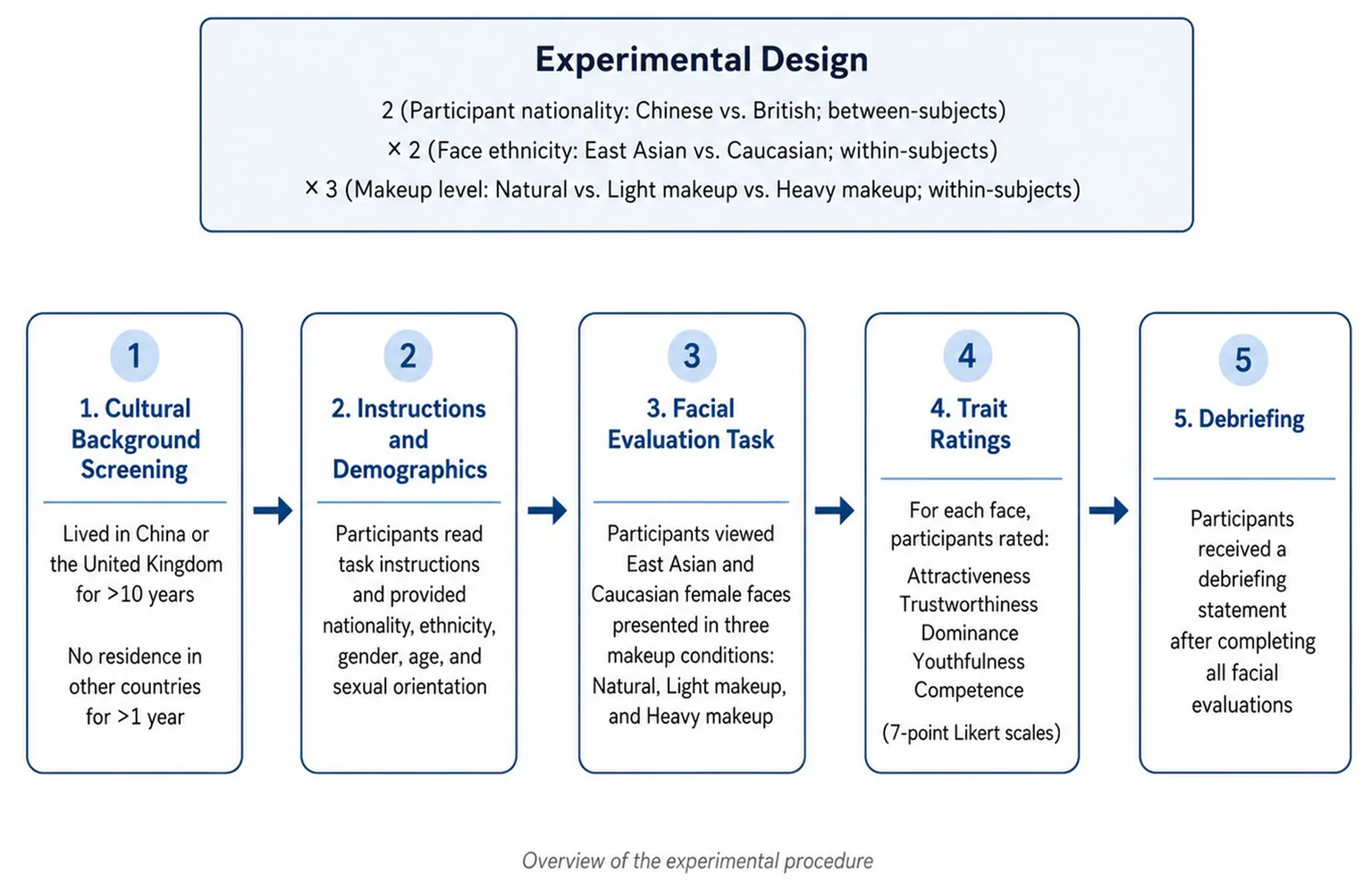
The examples of facial stimulus (Lundqvist et al., 1998).

The study employed 12 natural East Asian female faces obtained from the Generate photo website (https://generated.photos/faces) and 12 natural Caucasian female faces from Kdef facial database^3^. All faces were frontal-view photographs, with neutral expressions and direct gaze. Light and heavy makeup conditions were generated using the Meitu software, following procedures from previous research (Tagai et al., 2016). Examples of stimuli can be seen in Figure 1.

For the light makeup condition, foundation was blended smoothly into the skin tone. Facial feature outlines were softened, and the eyebrows were smoothed to create a gentle appearance. Eyeshadow was blended seamlessly with the skin tone, lips were tinged with a reddish gloss, and a soft red blush was applied to the cheeks, blending from the center outward.

For the heavy makeup condition, a matte foundation covered the skin, and facial features were emphasized with defined, straight lines. Eyebrows were drawn dark with sharp, straight lines to elevate the center of the brow. Dark eyeshadow was applied to produce elongated, slit-like eyes, and the lips were painted with a matte brownish color with sharp outlines. Importantly, the manipulation was designed to reflect ecologically realistic cosmetic styles rather than isolating a single visual feature. As in real-world cosmetic enhancement, differences between light and heavy makeup involved coordinated changes in facial contrast, color intensity, and feature definition.

Prior to the experiment, 25 participants rated the makeup intensity of all faces on a 7-point Likert scale, where 1 indicated minimal or no makeup and 7 indicated very heavy makeup. ANOVA confirmed a significant effect of makeup condition on perceived makeup intensity, *F* (2, 72) = 175.42, *p* < 0.001, partial *η*^2^ = 0.82. Post-hoc Tukey tests showed that faces in the heavy makeup condition (*M* = 6.13, SD = 0.87) were rated significantly higher than those in the light makeup condition (*M* = 3.52, SD = 0.95), *t* (72) = 10.91, *p* < 0.001) and faces in the light makeup condition were rated significantly higher than those in the natural condition (*M* = 1.85, SD = 0.73), *t* (72) = 7.73, *p* < 0.001).

### Procedure

The experimental procedures for both Chinese and British versions were implemented using the Credamo platform. Participants first completed a cultural background screening survey, responding to two questions: (1) “Have you lived in China/the United Kingdom for more than 10 years?” and (2) “Have you lived in any other country for more than 1 year?” Only participants who answered “yes” to the first question and “no” to the second proceeded to the main experiment.

Next, participants read the instructions and provided demographic information, including race, nationality, gender, age, and sexual orientation. Participants then evaluated each face on perceived attractiveness, perceived trustworthiness, perceived dominance, perceived youthfulness, and perceived competence. After all faces had been evaluated, participants were presented with a debriefing statement clarifying the overall purpose of the study.

### Data analysis

A linear mixed-effects model was conducted using the *lme4* package (Bates et al., 2015) in R to analyze the effect of all the fixation factors on attractiveness, youth, trustworthiness, competence and dominance. Akaike information criterion (AIC) was used as standard to select the best-fit model (Akaike, 1973). The model with the lowest AIC value was selected as the best-fit model and used for further analyses. The *p*-values of the parameter estimates for each model were obtained using the Satterthwaite approximation used by the *lmerTest* package (Kuznetsova et al., 2017). *Post hoc* pairwise comparisons were performed using the *lsmeans* package (Lenth, 2016). Results are reported for regression coefficients (*β*), standard errors (SEs), and *t*-values. Graphs were generated using the *ggplot2* package (Wickham, 2016).

## Results

### Perceived attractiveness

A linear mixed-effects model was conducted to examine the effects of makeup level (natural|light|heavy), face ethnicity (East Asian|Caucasian), and participant nationality (Chinese|British) on perceived facial attractiveness. Participant and stimuli were included as random intercepts.

Significant main effects were observed for both makeup and face ethnicity. Faces with natural (*β* = −0.63, SE = 0.16, *p* < 0.001) and light makeup (*β* = −0.35, SE = 0.16, *p* = 0.03) were rated as less attractive compared to faces with heavy makeup. Additionally, East Asian faces were rated as more attractive than Caucasian faces (*β* = 0.52, SE = 0.16, *p* = 0.002).

**Figure 2:**
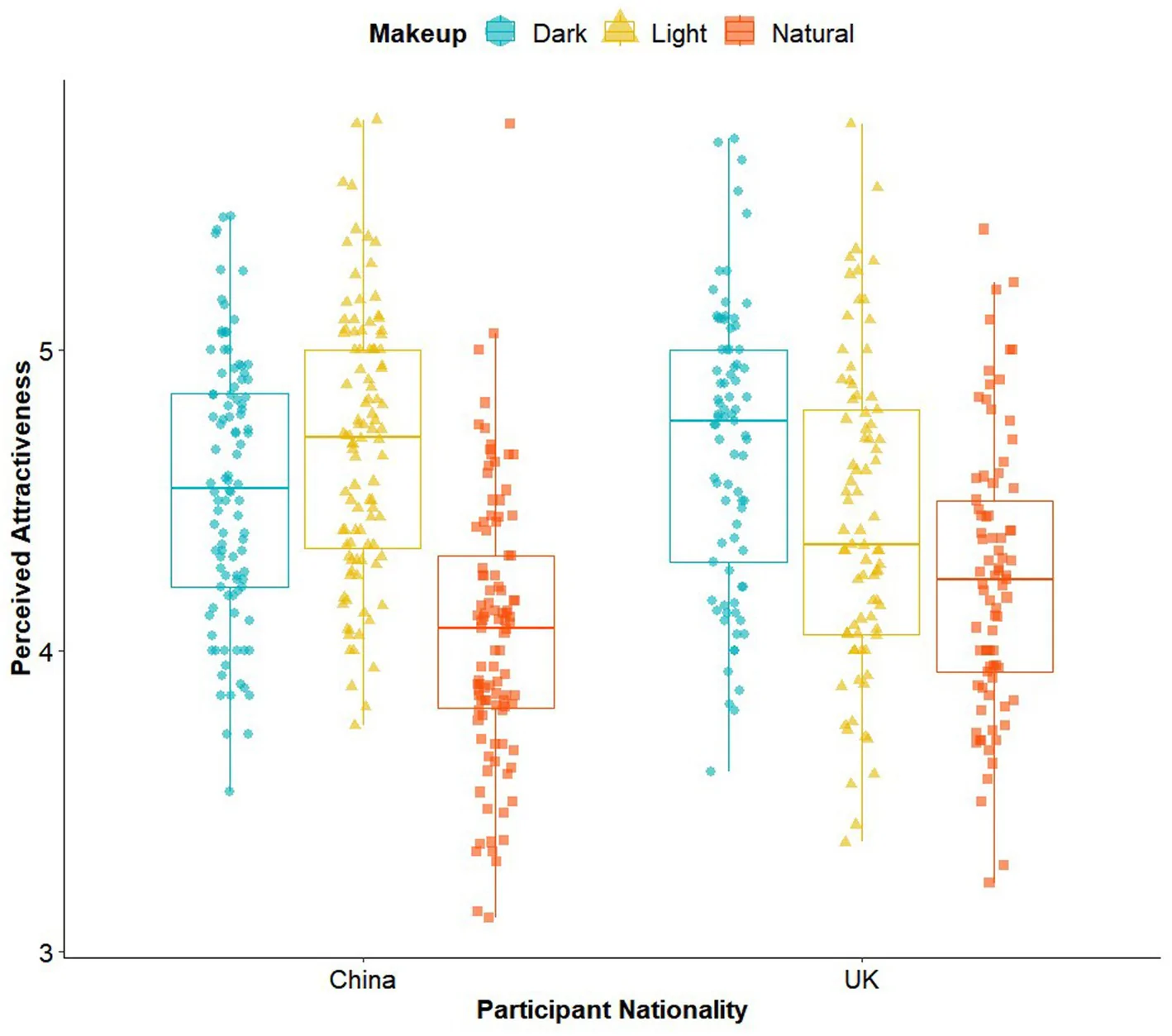
The interaction between makeup and participant nationality on perceived attractiveness.

The three-way interaction among makeup, face ethnicity, and participant nationality was not significant (all *p*s > 0.12). Two two-way interactions, however, reached significance. First, the interaction between makeup and participant nationality was significant, *β* = 0.41, SE = 0.06, *t* (8664) = 6.77, *p* < 0.001 (see Figure 2). Simple effects analyses revealed that Chinese participants rated light makeup (vs. natural) as more attractive (*β* = 0.71, SE = 0.11, *z* = 6.25, *p* < 0.001), but showed no significant difference between heavy and light makeup (*β* = 0.14, SE = 0.11, *z* = 1.26, *p* = 0.41). In contrast, British participants rated both heavy makeup as more attractive than natural (*β* = 0.51, SE = 0.12, *z* = 4.46, *p* < 0.001) and light makeup (*β* = 0.27, *SE* = 0.12, *z* = 2.39, *p* = 0.04), but no significant differences between natural and light makeup (*β* = 0.23, SE = 0.12, *z* = 2.06, *p* = 0.09).

**Figure 3:**
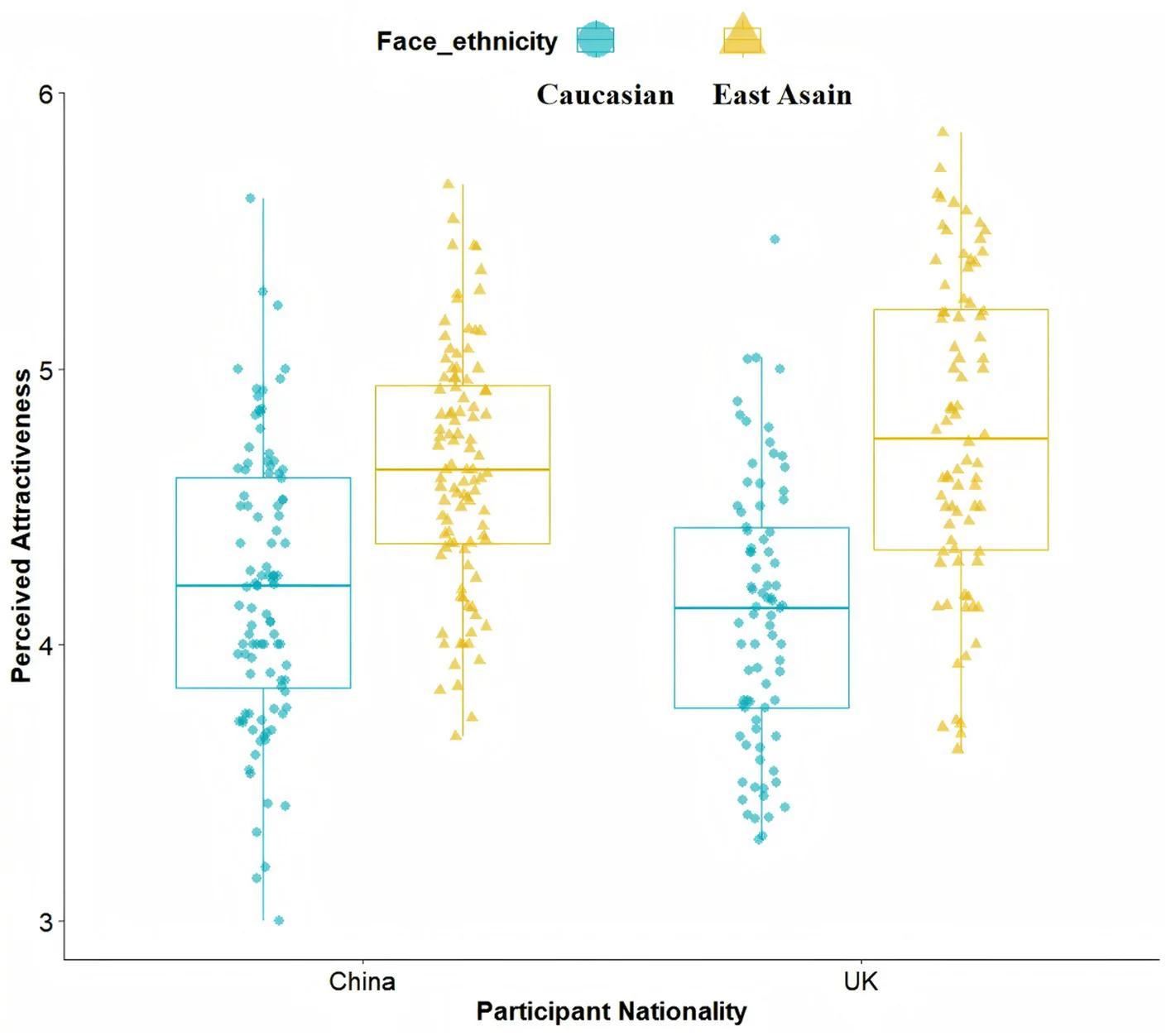
The interaction between face ethnicity and participant nationality on perceived attractiveness.

Second, the interaction between face ethnicity and participant nationality was significant (see Figure 3). Simple effects analyses indicated that Chinese participants rated East Asian faces as significantly less attractive than Caucasian faces (*β* = −0.50, SE = 0.09, *z* = 5.41, *p* < 0.001), whereas British participants exhibited the opposite pattern, rating East Asian faces as more attractive than Caucasian faces (*β* = 0.72, SE = 0.09, *z* = 7.72, *p* < 0.001).

### Perceived trustworthiness

A linear mixed-effects model was conducted to examine the effects of makeup level (natural|light|heavy), face ethnicity (East Asian|Caucasian), and participant nationality (Chinese|British) on perceived trustworthiness. A significant main effect was found for face ethnicity: East Asian faces were rated as more trustworthy than Caucasian faces (*β* = 0.63, SE = 0.10, *p* < 0.001). No significant main effects were found for makeup or participant nationality (*p*s > 0.20). The three-way interaction among makeup, face ethnicity, and participant nationality was not significant (*p*s > 0.23).

**Figure 4:**
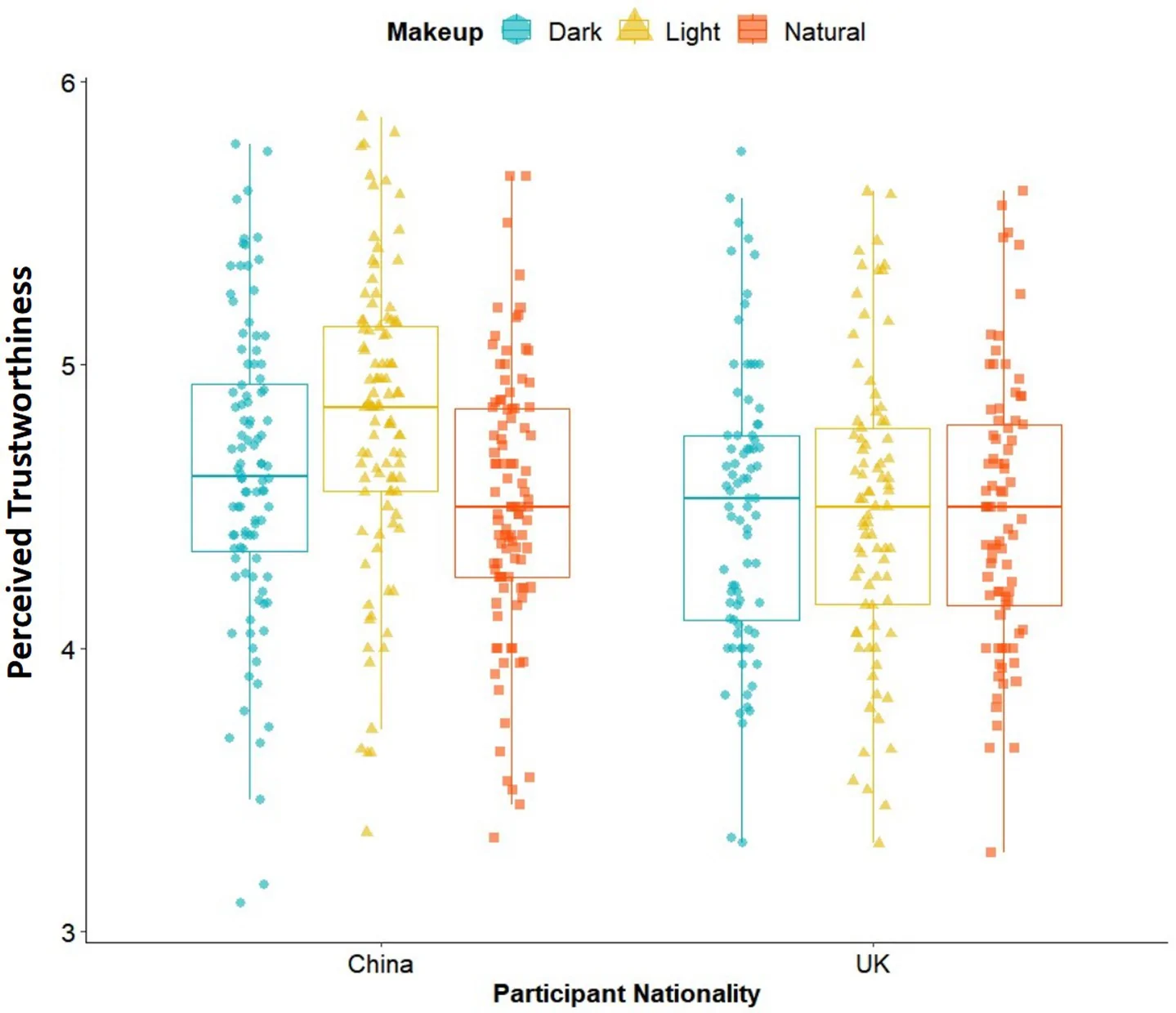
The interaction effect of makeup and participant nationality on perceived trustworthiness.

However, several two-way interactions emerged. First, the interaction between makeup and participant nationality was significant, *β* = −0.21, SE = 0.06, *t* (9367.30) = −3.70, *p* < 0.001 (see Figure 4). Simple effects analysis showed that Chinese participants rated light makeup as more trustworthy (*β* = 0.21, *p* < 0.001) than natural makeup (*β* = 0.33, SE = 0.07, *z* = 4.45, *p* < 0.001) and heavy makeup (*β* = 0.21, SE = 0.07, *z* = 2.77, *p* = 0.01), but no significant differences between heavy makeup and natural faces (*β* = 0.13, SE = 0.07, *z* = 1.69, *p* = 0.21). In contrast, British participants showed no significant differences in trustworthiness ratings across makeup levels (*p*s > 0.89). The interaction between face ethnicity and participant nationality was not significant (*p*s > 0.99), nor was the interaction between makeup and face ethnicity (*p*s > 0.26).

### Perceived dominance

A linear mixed-effects model was conducted to examine the effects of makeup level (natural|light|heavy), face ethnicity (East Asian|Caucasian), and participant nationality (Chinese|British) on perceived dominance. Random intercepts were included for participants and stimuli. The main effect of makeup was significant, *β* = −0.16, SE = 0.06, *t* (78.88) = −2.36, *p* = 0.02, with light makeup (*β* = 0.11, SE = 0.04, *z* = 2.57, *p* = 0.02) and natural faces (*β* = 0.16, SE = 0.04, *z* = 3.69, *p* < 0.001) rated as less dominant than heavy makeup, but there are not significant differences between light makeup and natural faces. No significant main effects were observed for face ethnicity (*β* = −0.01, SE = 0.07, *p* = 0.89) or participant nationality (*β* = −0.09, SE = 0.08, *p* = 0.27).

**Figure 5:**
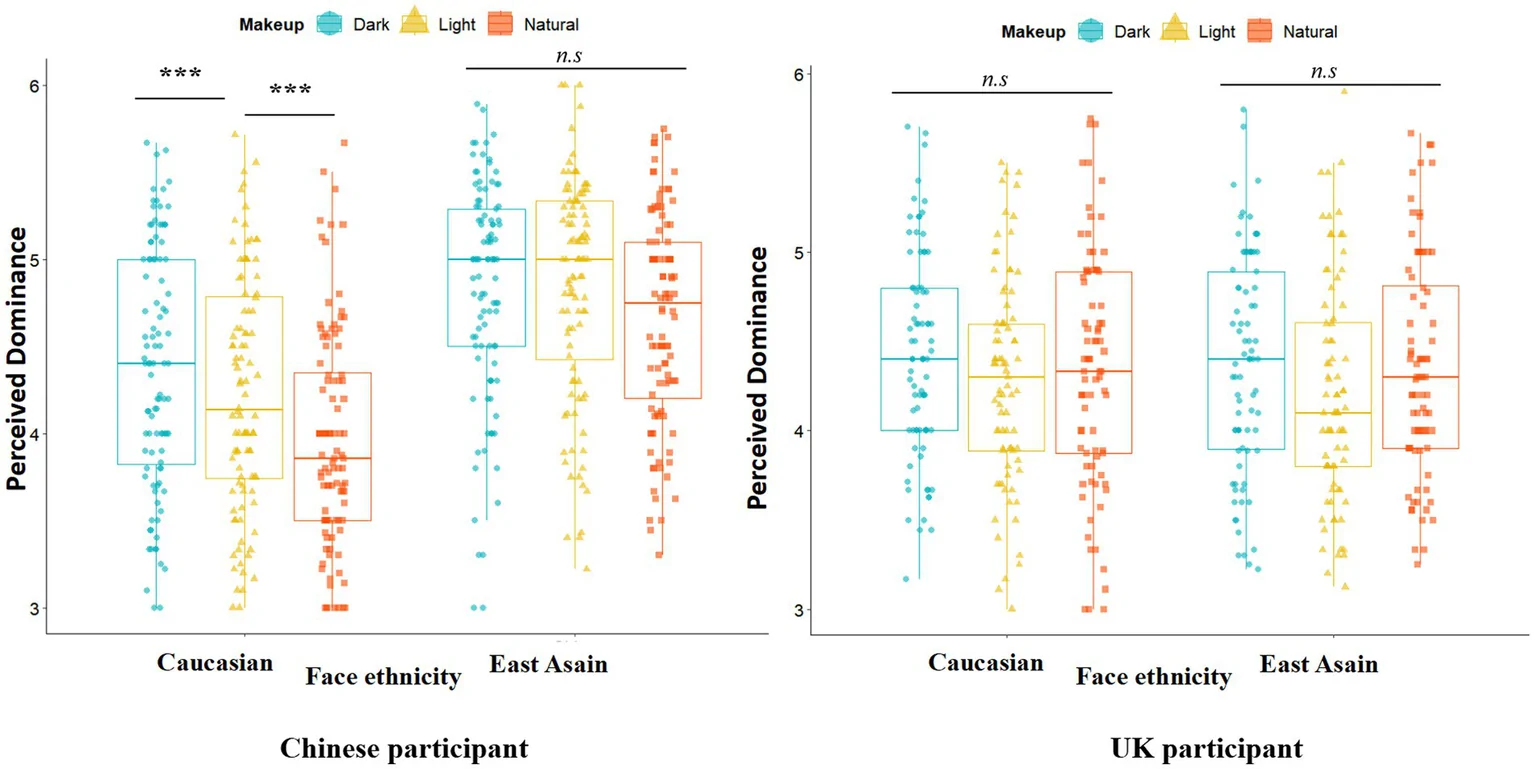
The three-way interaction of perceived dominance across makeup, participant ethnicity and face ethnicity.

The three-way interaction among makeup, face ethnicity, and participant nationality was significant (*β* = 0.22, SE = 0.08, *t* (9152.64) = 2.51, *p* = 0.012, see Figure 5). Among Chinese participants, when evaluating Caucasian faces, dominance ratings were lowest for natural faces, heavy makeup (*β* = 0.41, SE = 0.07, *z* = 5.95, *p* < 0.001) and light makeup (*β* = 0.25, SE = 0.07, *z* = 3.61, *p* < 0.004) significantly higher than natural faces. For East Asian faces, no significant effects were found for Chinese participants (*p*s > 0.10).

In contrast, British participants showed no significant differences in dominance ratings across makeup levels for either Caucasian or East Asian faces (all *p*s > 0.27). Similarly, dominance ratings did not differ significantly between Caucasian and East Asian faces within British participants.

### Perceived youthfulness

A linear mixed-effects model was conducted to examine the effects of makeup level (natural|light|heavy), face ethnicity (East Asian|Caucasian), and participant nationality (Chinese|British) on perceived youthfulness. Participants and facial stimuli were modeled as random intercepts. The main effect of makeup was significant, *β* = −0.87, SE = 0.14, *t* (58.24) = −6.16, *p* < 0.001, with light makeup (*β* = 0.68, SE = 0.09, *z* = 6.88, *p* < 0.001) and heavy makeup faces (*β* = 0.54, SE = 0.09, *z* = 5.51, *p* < 0.001) rated as younger than natural faces, but there are not significant differences between light makeup and heavy faces (*β* = 0.13, SE = 0.09, *z* = 1.37, *p* = 0.35). No significant main effects were observed for face ethnicity (*β* = −0.13, SE = 0.14, *p* = 0.32) or participant nationality (*β* = −0.09, SE = 0.07, *p* = 0.21).

**Figure 6:**
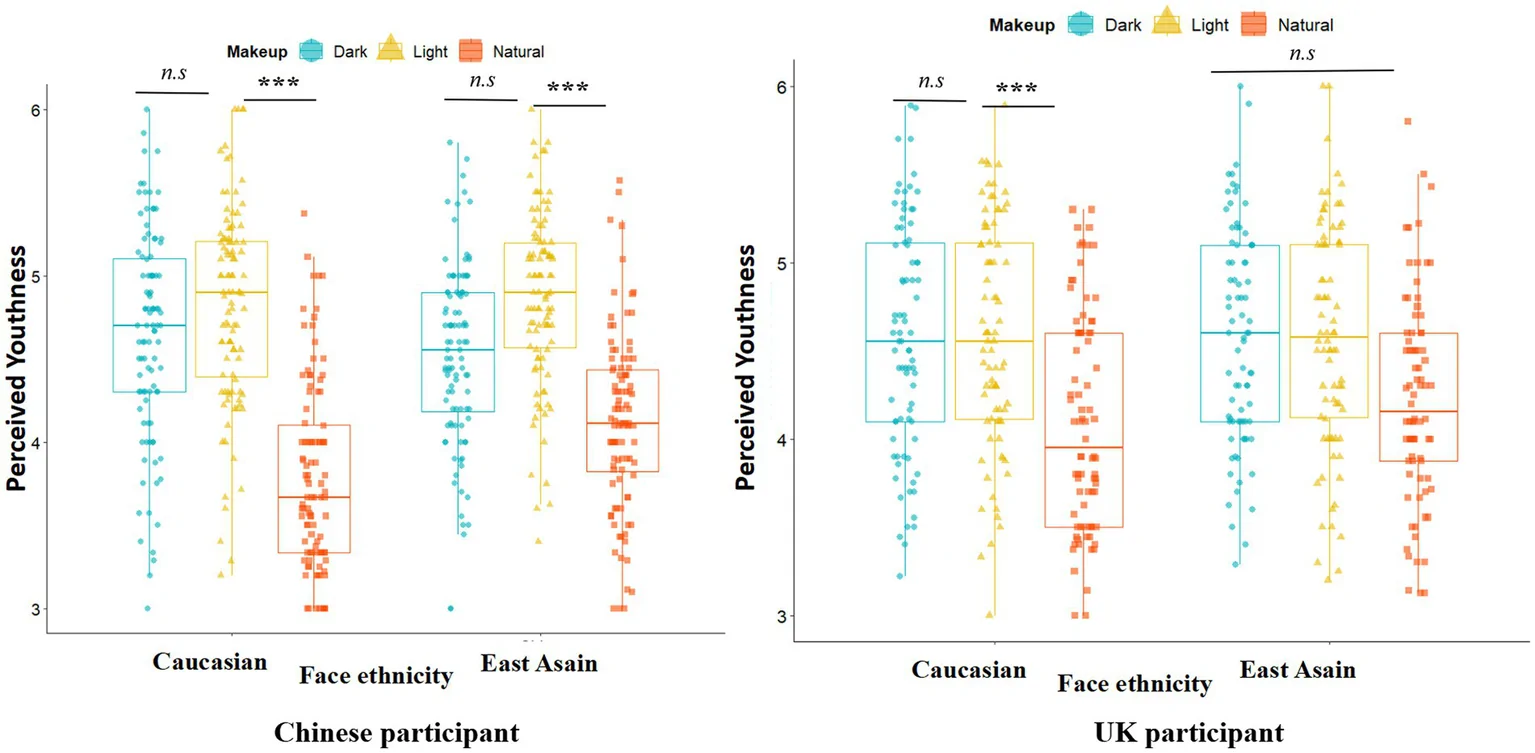
The three-way interaction of perceived youthness across makeup, participant ethnicity and face ethnicity.

A significant three-way interaction among makeup level, face ethnicity, and participant nationality emerged (*β* = 0.33, SE = 0.08, *t* (9123) = 3.92, *p* < 0.001). Among Chinese participants, facial makeup exerted a strong influence on perceived youthfulness, with similar patterns for both British and East Asian faces (see Figure 6). For both light makeup (Caucasian faces: *β* = 1.06, *p* < 0.001; East Asian faces: *β* = 0.74, *p* < 0.001) and heavy makeup (Caucasian faces: *β* = 0.88, *p* < 0.001; East Asian faces: *β* = 0.56, *p* = 0.001) were perceived as more youthful than natural makeup. Overall, Chinese participants consistently associated makeup, regardless of face ethnicity, with increased youthfulness and rated natural makeup as the least youthful.

Moreover, British participants rated both light makeup (*β* = 0.53, *p* = 0.002) and heavy makeup (*β* = 0.53, *p* = 0.002) as more youthful than natural makeup for Caucasian faces, with no significant difference between light and heavy makeup (*p* > 0.90). In contrast, for East Asian faces, no significant differences in perceived youthfulness emerged across makeup conditions (all *p*s > 0.09), suggesting that makeup exerted weaker effects on British participants’ youthfulness judgments of East Asian faces.

### Perceived competence

We fitted a linear mixed-effects model to examine the effects of makeup level, face ethnicity, and participant nationality on perceived competence, with random intercepts for participants and stimuli. A significant main effect of makeup emerged, *β* = −0.35, SE = 0.07, *t* (68.74) = −4.56, *p* < 0.001. Faces with natural makeup were rated as less capable than those with heavy makeup (*β* = 0.16, SE = 0.05, *z* = 3.19, *p* = 0.004) and light makeup (*β* = 0.13, SE = 0.05, *z* = 2.52, *p* = 0.03). Face ethnicity showed a robust effect, *β* = 0.50, SE = 0.07, *t* (67.75) = 6.64, *p* < 0.001, with East Asian faces judged as more capable than Caucasian faces. Participant nationality had no main effect (*β* = 0.02, SE = 0.08, *p* = 0.80).

**Figure 7:**
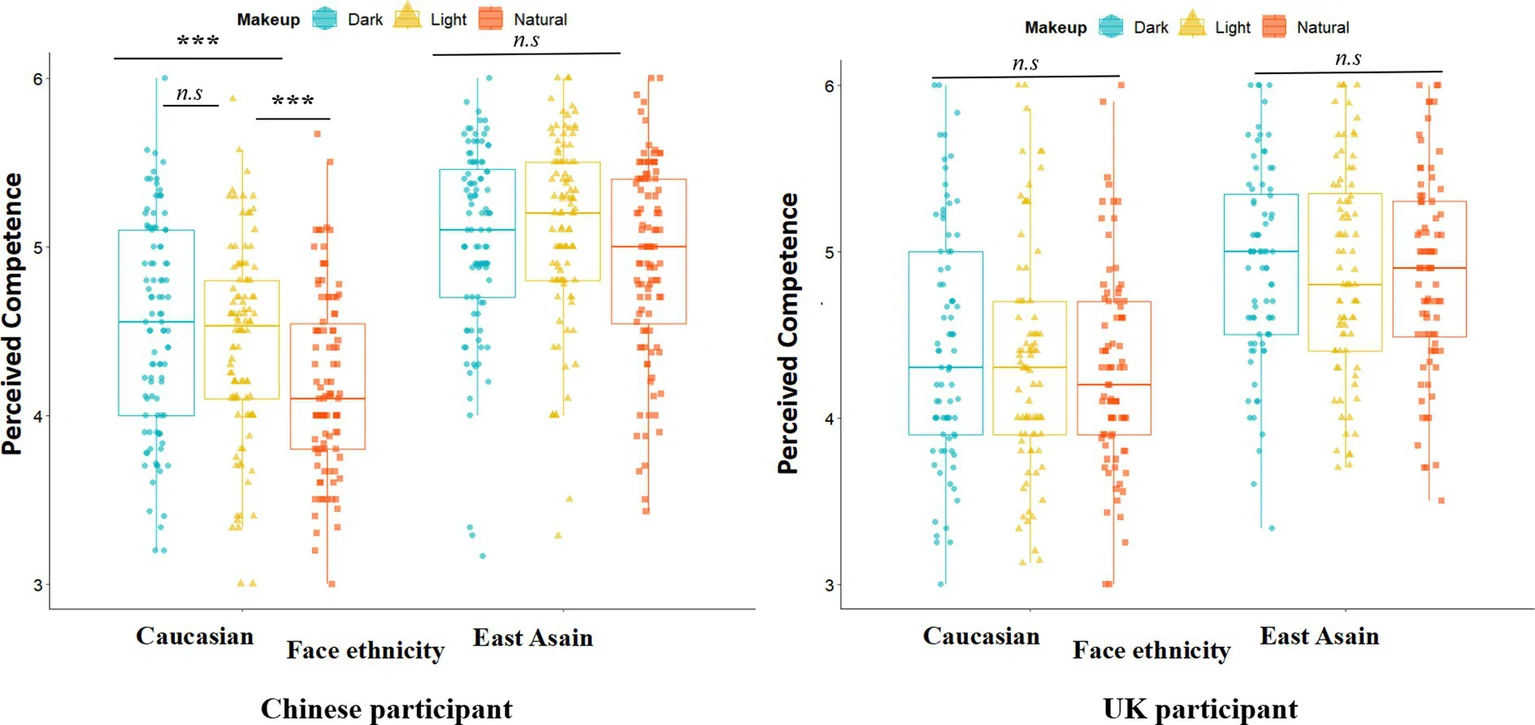
The three-way interaction of perceived competence across makeup, participant ethnicity and face ethnicity.

A significant three-way interaction was observed among makeup level, face ethnicity, and participant nationality (*β* = −0.17, SE = 0.07, *t* (9456.86) = −2.23, *p* = 0.02, see Figure 7). For Chinese participants, natural Caucasian faces received significantly lower competence ratings compared to the same faces with heavy makeup (*β* = −0.35, SE = 0.07, *z* = 4.56, *p* < 0.001) or light makeup (*β* = −0.28, SE = 0.08, *z* = 3.81, *p =* 0.002), but there were no significant differences between heavy and light makeup. For East Asian faces, no significant effects of makeup were found (*p*s > 0.18). For British participants, makeup level did not significantly influence competence ratings for either Chinese (heavy vs. natural: *p* = 0.16) or Caucasian faces (heavy vs. natural: *p* = 0.32).

## Discussion

The present study examined whether cosmetic enhancement merely increases facial attractiveness or more broadly reshapes social evaluation. The findings suggest that cosmetic enhancement does more than increase perceived attractiveness. Instead, cosmetic enhancement systematically altered judgments of attractiveness, competence, dominance, youthfulness, and, under some conditions, trustworthiness.

Makeup generally strengthened impressions linked to attractiveness and status, whereas its influence on trustworthiness was more contingent and differed across perceiver groups. Several effects also varied as a function of perceiver nationality and target ethnicity, suggesting that the evaluative consequences of cosmetic enhancement depend on who is evaluating whom.

Taken together, these findings suggest that makeup-induced appearance cues can operate as social signals in facial impression formation, shaping multiple dimensions of facial perception beyond beauty.

### Cosmetic enhancement shapes socially valued impressions

The present findings provide substantial support for the view that cosmetic enhancement amplifies socially valued impressions rather than merely increasing perceived beauty. Across conditions, makeup reliably increased judgments of attractiveness and youthfulness, a pattern that is consistent with prior evidence showing that cosmetics enhance facial contrast and accentuate appearance cues associated with femininity and attractiveness. Russell (2009) showed that facial contrast is sexually dimorphic and that cosmetics exaggerate this contrast, whereas Etcoff et al. (2011) further demonstrated that cosmetics can enhance the perception of biologically important facial signals, including attractiveness. More recent work has likewise shown that faces with cosmetics are often judged as more attractive than natural faces, although the perceived benefits of enhancement may depend on makeup intensity and viewing context (Etcoff et al., 2011; Tagai et al., 2016; Jones and Kramer, 2016; Aguinaldo and Peissig, 2021).

Importantly, the present results indicate that these amplification effects extend beyond attractiveness-related judgments. Makeup also increased competence and, in several conditions, dominance, suggesting that cosmetic enhancement can influence broader inferences about social capability and interpersonal impact.

This interpretation is in line with prior research showing that women wearing makeup are often perceived as more competent in professional or leadership-relevant contexts, and that cosmetics can also alter impressions related to prestige and dominance. Klatt et al. (2016), for example, found that makeup increased perceived competence, and Mileva et al. (2016) reported that cosmetics shaped judgments of women’s dominance and prestige.

Similarly, Aguinaldo and Peissig (2021) found that faces with cosmetics were judged as more competent than faces without cosmetics. Taken together, these converging findings suggest that cosmetic enhancement does not simply beautify the face; it also increases the salience of cues that observers appear to associate with capability, and social presence.

From this perspective, cosmetic enhancement may be understood as a socially meaningful appearance cue that strengthens multiple positive impressions at once. In the present study, attractiveness and youthfulness were enhanced relatively consistently, whereas competence and dominance also shifted in the expected direction, though more selectively across perceiver groups and target categories. This overall pattern supports the view that appearance enhancement is not merely an esthetic modification, but can operate as a social signal in facial impression formation by shaping impressions of attractiveness, youthfulness, competence, and dominance.

### Trustworthiness as a context-sensitive dimension of cosmetic evaluation

Although cosmetic enhancement generally amplified attractiveness- and status-related impressions, its influence on trustworthiness followed a more conditional pattern. In the present study, lightly made-up faces were judged as more trustworthy than natural or heavily made-up faces among Chinese participants, whereas British participants showed no systematic trustworthiness differences across makeup conditions. This finding suggests that the consequences of cosmetic enhancement for trust-based judgments may not follow a simple linear pattern.

Previous research has similarly indicated that different dimensions of social evaluation do not respond uniformly to cosmetic enhancement. Cosmetics reliably increase attractiveness by enhancing facial contrast and accentuating sexually dimorphic facial features (Russell, 2009).

Consistent with this pattern, Etcoff et al. (2011) showed that while cosmetics consistently increased attractiveness, their effects on other social judgments, such as likability and trustworthiness, depended on the specific cosmetic style and evaluation conditions. In particular, more dramatic cosmetic looks produced lower trustworthiness ratings under rapid judgments, suggesting that different evaluative dimensions may respond differently to appearance enhancement.

However, the effects of cosmetics on broader social judgments appear to be more nuanced. While makeup can increase perceptions of competence or prestige in some contexts (Aguinaldo and Peissig, 2021; Mileva et al., 2016), other work suggests that appearance enhancement may also introduce ambiguity regarding sincerity or authenticity.

Samper et al. (2018) showed that highly effortful beauty work can trigger inferences of misrepresentation and reduce judgments of moral character, and Mittal and Silvera (2020) reported that heavier makeup decreased perceived trustworthiness of salespeople. These findings suggest that while cosmetic enhancement may strengthen cues linked to attractiveness or status, it can also alter observers’ interpretations of authenticity or intention.

When enhancement is subtle, it may signal effort or social polish without raising concerns about strategic modification. Rather than observing a simple decrease in trustworthiness with increasing makeup, we found that moderate enhancement sometimes produced the most favorable trust judgments.

One possible interpretation is that trustworthiness evaluations may be particularly sensitive to the perceived balance between enhancement and naturalness. When enhancement is subtle, it may convey effort or social polish without raising concerns about artificiality or excessive self-presentation. When enhancement becomes more pronounced, however, observers may interpret the appearance as more artificial or deliberate, which could weaken trust-related impressions.

Importantly, the fact that this pattern emerged primarily among Chinese participants suggests that observers from different cultural contexts may differ in how strongly cosmetic cues influence trust-based evaluations.

### Cultural variation in the interpretation of cosmetic signals

A further set of findings concerns cultural and intergroup variation in the effects of cosmetic enhancement. Although makeup generally increased attractiveness-related impressions, its effects on competence, dominance, youthfulness, and trustworthiness differed depending on perceiver nationality and target ethnicity. These findings suggest that makeup-induced appearance cues may not carry the same evaluative meaning across all perceiver-target contexts.

This interpretation is broadly consistent with cross-cultural work showing that facial first impressions are not organized identically across perceiver groups (Sutherland et al., 2018). Extending this perspective, our findings show that the effects of cosmetic enhancement on specific trait judgments vary across Chinese and British perceivers and across East Asian and Caucasian faces.

The attractiveness findings also caution against a simple own-race preference interpretation. Chinese participants rated Caucasian faces as more attractive than East Asian faces, whereas British participants rated East Asian faces as more attractive than Caucasian faces. Prior work suggests that facial attractiveness judgments often show substantial cross-cultural agreement, while still varying with target ethnicity and facial characteristics (Coetzee et al., 2014; Little et al., 2011). Recent work on personal beauty values also suggests that beauty-related judgments involve dimensions such as social appropriateness, cultural flawlessness, self-improvement, and natural uniqueness, which may vary across cultural contexts (Glückstad et al., 2025).

The present cross-ethnicity pattern may therefore reflect multiple factors rather than a straightforward preference for out-group faces, including novelty or relative unfamiliarity, media exposure to globalized beauty ideals, attractiveness stereotypes, or baseline stimulus differences in cues such as averageness, sexual dimorphism, skin texture, or perceived age.

One possible mechanism underlying the trustworthiness findings concerns culturally shaped norms about cosmetic appropriateness. Prior research indicates that different makeup styles can have different social consequences. Etcoff et al. (2011) found that cosmetics increased attractiveness and competence, whereas judgments of likability and trustworthiness depended on the specific makeup look and viewing condition. Similarly, Tagai et al. (2016) showed that light makeup was evaluated more favorably than heavy makeup and suggested that heavy makeup may draw greater attention to the cosmetic style itself rather than the individual wearing it. These findings provide a useful context for interpreting why Chinese participants rated lightly made-up faces as more trustworthy than both natural and heavily made-up faces. Light makeup may convey social polish, self-care, or norm-congruent grooming, whereas heavier makeup may appear more deliberate or artificial.

Another possible mechanism concerns reliance on salient appearance cues when evaluating less familiar or out-group faces. Impression formation involves both category-based and individuating processes (Fiske and Neuberg, 1990), and cross-race face processing is shaped by social categorization, individuation motivation, and perceptual experience (Hugenberg et al., 2010; Young et al., 2012). These perspectives may help explain why Chinese participants were more influenced by cosmetics when evaluating Caucasian faces, particularly for judgments of dominance, competence, and youthfulness.

However, because the present study did not directly measure perceived makeup appropriateness, familiarity with cosmetic styles, intergroup contact, media exposure, novelty, or attractiveness stereotypes, these interpretations should be treated as plausible explanations rather than definitive mechanisms. Future work should directly assess these variables and use larger and more diverse face sets to clarify how cosmetic enhancement is interpreted across cultural and intergroup contexts.

### Theoretical contributions

The present findings make three theoretical contributions to research on cosmetic enhancement, facial social perception, and cross-cultural person evaluation. First, the study advances research on cosmetics by shifting the focus from whether makeup increases attractiveness to how cosmetic enhancement operates as a socially interpretable cue in facial impression formation. Previous work has shown that cosmetics enhance facial attractiveness by modifying features such as facial contrast, skin appearance, and other beauty-related cues (Russell, 2009; Etcoff et al., 2011; Batres et al., 2025; Sun et al., 2022). The present findings extend this work by showing that makeup-induced appearance cues also shape broader trait impressions, including youthfulness, competence, dominance, and trustworthiness. Cosmetic enhancement therefore should not be understood only as an esthetic intervention. It can also function as a social signal through which observers infer socially relevant traits from faces.

Second, the study clarifies that the social meaning of cosmetic enhancement contains both relatively general and context-dependent components. Attractiveness and youthfulness showed relatively consistent enhancement effects, suggesting that some consequences of makeup are closely tied to its visual beautification function. In contrast, competence, dominance, and especially trustworthiness showed more selective or conditional patterns across perceiver nationality, target ethnicity, and makeup intensity. This distinction helps specify which aspects of cosmetic evaluation are more likely to generalize across contexts and which aspects depend more strongly on how the enhancement is socially interpreted. In this sense, the present study moves beyond the broad claim that culture or social norms matter and instead shows that the same appearance-enhancement practice can carry different meanings depending on the trait being judged and the perceiver-target context in which the face is evaluated.

Third, the findings contribute to theories of face perception by demonstrating that social impressions from faces are shaped not only by relatively stable facial structure, but also by intentional and modifiable appearance cues. Classic models of face evaluation have emphasized dimensions such as trustworthiness, dominance, and attractiveness as rapid inferences derived from facial appearance (Oosterhof and Todorov, 2008; Todorov et al., 2015). The present study extends this perspective by showing that these impressions can be systematically altered through cosmetic enhancement, and that the interpretation of these altered cues varies across social and cultural contexts. This is theoretically important because faces in everyday life are rarely encountered as purely natural stimuli. They are often modified, styled, and socially curated. Understanding facial social perception therefore requires attention not only to stable facial features, but also to the appearance practices through which faces become socially meaningful.

### Limitations and future directions

Several limitations of the present research should be acknowledged. First, the manipulation of cosmetic enhancement necessarily involved coordinated changes in multiple visual features, including color intensity and facial contrast. In real-world cosmetic practice, heavier makeup typically entails stronger pigmentation and greater contrast across facial features (Russell, 2009). As a result, the present manipulation reflects ecologically realistic cosmetic styles rather than the isolated manipulation of a single visual parameter. Future research could experimentally disentangle specific components of cosmetic enhancement, such as color saturation, contrast, or contouring to examine how these visual elements independently contribute to social evaluations.

Second, the present study relied on static facial photographs and focused on two cultural populations. Although this approach allowed for controlled comparisons across perceiver nationality and target ethnicity, facial impressions in everyday social interaction are often formed under more dynamic conditions and within broader cultural contexts. Future studies could therefore examine cosmetic enhancement in dynamic stimuli, such as video or real-time interaction, and include a wider range of cultural groups to further clarify how appearance enhancement is interpreted across social contexts.

Third, the present study focused on observers’ trait judgments and therefore speaks primarily to cosmetic enhancement as social signaling in facial impression formation. Future work could extend this observer-centered approach by examining how cosmetic users adjust makeup across different social goals and whether observer impressions translate into downstream interpersonal decisions, such as mate choice, trust decisions, hiring evaluations, or status allocation. Such work would help clarify how makeup-induced appearance cues operate across the broader sequence from appearance modification to social perception and interpersonal behavior.

## Conclusion

The present research shows that cosmetic enhancement shapes facial social evaluation not simply by making faces appear more attractive, but by altering the social meanings that observers infer from faces. Across Chinese and British perceivers, makeup generally strengthened beauty-, vitality-, and status-related impressions, including attractiveness, youthfulness, competence, and dominance. However, these effects were not uniformly distributed across all evaluative domains. Trustworthiness judgments were more context-dependent, and several effects varied according to makeup intensity, perceiver cultural background, and target ethnicity.

These findings suggest that makeup-induced appearance cues can operate as social signals in facial impression formation. Some consequences of cosmetic enhancement appear relatively general, particularly those closely tied to visual beautification, whereas others depend more strongly on the social and cultural context in which the enhanced face is interpreted. More broadly, the present study highlights that the social consequences of appearance enhancement depend not only on how faces are modified, but also on what is being judged and who is evaluating whom.

## Data Availability

The raw data supporting the conclusions of this article will be made available by the authors, without undue reservation.

## Footnotes

1
https://osf.io/zjm2c

2
https://www.meituxiuxiu.com/

3
https://kdef.se/faq/using-and-publishing-kdef-and-akdef

## References

[ref1] Aguinaldo A. Peissig J. J. (2021). Cosmetics influence perception of female faces. Perception 50, 583–599. doi: 10.1177/03010066211021891

[ref2] Akaike H. (1973). Information theory and an extension of the maximum likelihood principle. In Petrov B. N. Csaki F. (eds.), Proceedings of the Second International Symposium on Information Theory (pp. 267–281). Budapest: Akademiai Kiado

[ref3] Appel M. Hutmacher F. Politt T. Stein J.-P. (2023). Swipe right? Using beauty filters in male tinder profiles reduces women’s evaluations of trustworthiness but increases physical attractiveness and dating intention. Comput. Hum. Behav. 148:107871. doi: 10.1016/j.chb.2023.107871

[ref4] Bates D. Mächler M. Bolker B. Walker S. (2015). Fitting linear mixed-effects models using lme4. J. Stat. Softw. 67, 1–48. doi: 10.18637/jss.v067.i01

[ref5] Batres C. Jones A. L. Barlett C. P. Porcheron A. Morizot F. Russell R. (2025). Makeup works by modifying factors of facial beauty. Psychol. Aesthet. Creat. Arts 19, 193–204. doi: 10.1037/aca0000505

[ref6] Bernard P. Content J. Deltenre P. Colin C. (2020). When attractiveness is not enough: beauty and trustworthiness in cosmetic use. Front. Psychol. 11:1876. doi: 10.3389/fpsyg.2020.01876 32849100 PMC7411309

[ref9004] Coetzee V. Greeff J. M. Stephen I. D. Perrett D. I. (2014). Cross-cultural agreement in facial attractiveness preferences: the role of ethnicity and gender. PLoS One 9:e99629. doi: 10.1371/journal.pone.0099629 24988325 PMC4079334

[ref7] Davis A. C. Arnocky S. (2022). An evolutionary perspective on appearance enhancement behavior. Arch. Sex. Behav. 51, 3–37. doi: 10.1007/s10508-020-01745-4, 33025291

[ref8] Etcoff N. Stock S. Haley L. Vickery S. House D. (2011). Cosmetics as a feature of the extended human phenotype: modulation of the perception of biologically important facial signals. PLoS One 6:e25656. doi: 10.1371/journal.pone.0025656, 21991328 PMC3185017

[ref9] Faul F. Erdfelder E. Buchner A. Lang A.-G. (2009). Statistical power analyses using G*power 3.1: tests for correlation and regression analyses. Behav. Res. Methods 41, 1149–1160. doi: 10.3758/BRM.41.4.1149 19897823

[ref10] Fiske S. T. Neuberg S. L. (1990). “A continuum of impression formation, from category-based to individuating processes: influences of information and motivation on attention and interpretation,” in Advances in Experimental Social Psychology, ed. Zanna M. P., vol. 23 (Cambridge, MA: Academic Press), 1–74.

[ref11] Glückstad F. K. Kobayashi H. Seddig D. Davidov E. Nakamura R. (2025). Personal beauty values: development and validation of a multidimensional measurement scale. J. Consum. Behav. 24, 282–303. doi: 10.1002/cb.2421

[ref12] Grafen A. (1990). Biological signals as handicaps. J. Theor. Biol. 144, 517–546. doi: 10.1016/S0022-5193(05)80088-8, 2402153

[ref15] Henrich J. Heine S. J. Norenzayan A. (2010). The weirdest people in the world? Behav. Brain Sci. 33, 61–83. doi: 10.1017/S0140525X0999152X, 20550733

[ref14] He X. Liang F. Wang X. Liu Y. Zhang J. Luo J. (2023b). Girls tolerate girls, men condemn men: gender differences in the impact of beautification strategies on trustworthiness and morality evaluation. Heliyon 9:e20365. doi: 10.1016/j.heliyon.2023.e20365, 37810802 PMC10550622

[ref13] He X. Li S. Zhang Y. (2023a). Facial trustworthiness judgments and social evaluation in Chinese observers. Front. Psychol. 14:1145297. doi: 10.3389/fpsyg.2023.1145297

[ref9006] Hugenberg K. Young S. G. Bernstein M. J. Sacco D. F. (2010). The categorization-individuation model: an integrative account of the other-race recognition deficit. Psychol. Rev. 117, 1168–1187. doi: 10.1037/a0020463 20822290

[ref16] James E. A. Jenkins S. Watkins C. D. (2018). Negative effects of makeup use on perceptions of leadership ability across two ethnicities. Perception 47, 540–549. doi: 10.1177/0301006618763263, 29523050

[ref9003] Jones A. L. Kramer R. S. S. (2016). Facial cosmetics and attractiveness: comparing the effect sizes of professionally-applied cosmetics and identity. PLoS One 11:e0164218. doi: 10.1371/journal.pone.0164218 27727311 PMC5058481

[ref17] Klatt J. Eimler S. C. Krämer N. C. (2016). Makeup your mind: The impact of styling on perceived competence and warmth of female leaders. The Journal of social psychology, 156, 483–497. doi: 10.1080/00224545.2015.1129303, 26852886

[ref18] Kowal M. Sorokowski P. Pisanski K. Valentova J. V. Varella M. A. Frederick D. A. . (2022). Predictors of enhancing human physical attractiveness: data from 93 countries. Evol. Hum. Behav. 43, 455–474. doi: 10.1016/j.evolhumbehav.2022.08.003

[ref19] Kuehl S. DeWild S. Mai J. Yeng Yang M. (2018). Is beauty only skin deep? Exploring the connections between makeup and perception. Concordia J Commun Res 5:1. doi: 10.54416/usgb5571

[ref20] Kuznetsova A. Brockhoff P. B. Christensen R. H. B. (2017). lmerTest package: tests in linear mixed effects models. J. Stat. Softw. 82, 1–26. doi: 10.18637/jss.v082.i13

[ref21] Lenth R. V. (2016). Least-squares means: the R package lsmeans. J. Stat. Softw. 69, 1–33. doi: 10.18637/jss.v069.i01

[ref22] Liang F. Zhu M. Lei Y. Zhang S. Liang M. Tao Z. . (2025). Untrustworthy beauty: beautification strategies cause female applicators to be perceived untrustworthy. Psychol. Aesthet. Creat. Arts 19:1219. doi: 10.1037/aca0000615

[ref23] Lin C. Keles U. Thornton M. A. Adolphs R. (2025). How trait impressions of faces shape subsequent mental state inferences. Nat. Hum. Behav. 9, 208–226. doi: 10.1038/s41562-024-02059-4, 39622977

[ref9005] Little A. C. Jones B. C. DeBruine L. M. (2011). Facial attractiveness: evolutionary based research. Philos. Trans. R. Soc. B Biol. Sci. 366, 1638–1659. doi: 10.1098/rstb.2010.0404 PMC3130383 21536551

[ref9010] Lundqvist D. Flykt A. Öhman A. (1998). The Karolinska Directed Emotional Faces—KDEF. CD-ROM, Department of Clinical Neuroscience, Psychology Section, Karolinska Institutet.

[ref24] Mileva V. R. Jones A. L. Russell R. Little A. C. (2016). Sex differences in the perceived dominance and prestige of women with and without cosmetics. Perception 45, 1166–1183. doi: 10.1177/0301006616652053, 27288188

[ref25] Mittal B. Silvera D. H. (2020). Does makeup influence perceptions of trustworthiness? Evidence from consumer judgments. J. Consum. Behav. 19, 241–252. doi: 10.1002/cb.1808

[ref26] Oosterhof N. N. Todorov A. (2008). The functional basis of face evaluation. Proc. Natl. Acad. Sci. 105, 11087–11092. doi: 10.1073/pnas.0805664105, 18685089 PMC2516255

[ref27] Russell R. (2009). A sex difference in facial contrast and its exaggeration by cosmetics. Perception 38, 1211–1219. doi: 10.1068/p6331 19817153

[ref28] Samper A. Yang Z. Daniels M. (2018). Beauty as a double-edged sword: the role of attractiveness in moral judgments. J. Consum. Res. 45, 1006–1026. doi: 10.1093/jcr/ucy042

[ref29] Sun Y.-H. P. Zhang X. Lu N. Li J. Wang Z. (2022). Your face looks the same as before, only prettier: the facial skin homogeneity effects on face change detection and facial attractiveness perception. Front. Psychol. 13:935347. doi: 10.3389/fpsyg.2022.935347, 36405180 PMC9667065

[ref9001] Sutherland C. A. M. Liu X. Zhang L. Chu Y. Oldmeadow J. A. Young A. W. (2018). Facial first impressions across culture: data-driven modeling of Chinese and British perceivers’ unconstrained facial impressions. Pers. Soc. Psychol. Bull. 44, 521–537. doi: 10.1177/0146167217744194 29226785

[ref30] Tagai K. Ohtaka H. Nittono H. (2016). Faces with light makeup are better recognized than faces with heavy makeup. Front. Psychol. 7:226. doi: 10.3389/fpsyg.2016.00226, 26973553 PMC4771839

[ref31] Todorov A. Olivola C. Y. Dotsch R. Mende-Siedlecki P. (2015). Social attributions from faces: determinants, consequences, and accuracy. Annu. Rev. Psychol. 66, 519–545. doi: 10.1146/annurev-psych-113011-143831 25196277

[ref32] Todorov A. Said C. P. Engell A. D. Oosterhof N. N. (2008). Understanding evaluation of faces on social dimensions. Trends Cogn. Sci. 12, 455–460. doi: 10.1016/j.tics.2008.10.001, 18951830

[ref33] Vorauer J. D. Turpie C. A. (2004). Disruptive effects of vigilance on dominant group members’ treatment of outgroup members. J. Pers. Soc. Psychol. 87, 384–399. doi: 10.1037/0022-3514.87.3.384 15382987

[ref9002] Wang H. Han C. Hahn A. C. Fasolt V. Morrison D. K. Holzleitner I. J. . (2019). A data-driven study of Chinese participants’ social judgments of Chinese faces. PLoS One 14:e0210315. doi: 10.1371/journal.pone.0210315 30608990 PMC6319767

[ref34] Wickham H. (2016). ggplot2: Elegant Graphics for Data Analysis. Berlin: Springer.

[ref35] Willis J. Todorov A. (2006). First impressions: making up your mind after a 100-ms exposure to a face. Psychol. Sci. 17, 592–598. doi: 10.1111/j.1467-9280.2006.01750.x, 16866745

[ref9007] Young S. G. Hugenberg K. Bernstein M. J. Sacco D. F. (2012). Perception and motivation in face recognition: a critical review of theories of the cross-race effect. Pers. Soc. Psychol. Rev. 16, 116–142. doi: 10.1177/1088868311418987 21878608

